# Clinical characteristics and risk factors of bacillary layer detachment in central serous chorioretinopathy: a comparative multicenter study

**DOI:** 10.1186/s40942-024-00612-x

**Published:** 2024-12-18

**Authors:** Antonio M. Casella, Raphaela M. Fuganti, Ahmad M. Mansour, José Ignácio Fernández-Vigo, Suthasinee Sinawat, Ali Osman Saatci, Jay Chhablani, Arman Zarnegar, Juliana Prazeres, Igor Kozak, Lorenzo López Guajardo, Michel E. Farah, Claudio Zett, Francisco Rodriguez, William E. Smiddy, Janet L. Davis, Philip J. Rosenfeld, Stephen G. Schwartz, Luiz H. Lima, Maurício Maia

**Affiliations:** 1 Londrina State University, Avenida Robert Koch, 60, Londrina, Paraná Brazil; 2 Federal University of São Paulo, São Paulo, Brazil; 3 American University of Beirut, Beirut, Lebanon; 4 Hospital Clínico San Carlos, Instituto de Investigación Sanitaria, Madrid, Spain; 5 Srinagarind Hospital, Khon Kaen University, Khon Kaen, Thailand; 6 Dokuz Eylul University School of Medicine, Izmir, Turkey; 7 University of Pittsburgh School of Medicine, Pittsburgh, PA USA; 8 Research Department, Moorfields Eye Hospital Center, Abu Dhabi, United Arab Emirates; 9 Pontificia Universidad Católica de Valparaíso, Valparaíso, Chile; 10 Fundación Oftalmológica Nacional, Universidad del Rosario, Bogotá, Colombia; 11 Bascom Palmer Eye Institute, Miami, FL USA

**Keywords:** Central serous chorioretinopathy, Bacillary layer detachment, Photodynamic therapy, Inflammation

## Abstract

**Background:**

Central serous chorioretinopathy (CSC) is marked by serous retinal detachments caused by fluid leakage from the retinal pigment epithelium, often associated with stress, psychiatric disorders and the use of corticosteroids. This study aims to investigate the clinical and systemic characteristics associated with BALAD in patients with CSC, comparing those with and without BALAD to clarify its function as a biomarker of CSC severity and improve diagnostic and treatment approaches.

**Purpose:**

Compare the clinical characteristics, risk factors, and optical coherence tomography (OCT) findings in patients with Central Serous Chorioretinopathy (CSC) with and without Bacillary Layer Detachment (BALAD), and to identify the distinguishing features and associated conditions of CSC with BALAD.

**Methods:**

This observational, retrospective, multicenter case–control study collected data from 12 retina centers worldwide on patients with central serous chorioretinopathy (CSC) from December 1, 2022, to April 1, 2023. CSC was defined by serous retinal detachment and fluid leakage through the retinal pigment epithelium. Patients underwent detailed evaluations, including OCT, and were classified as having acute or chronic CSC. Inclusion criteria included a CSC diagnosis with RPE leakage, BALAD confirmed by three authors, age over 18, and a detailed medical history from the 30 days before symptom onset. The study assessed visual acuity, choroidal thickness, psychiatric disorders, corticosteroid use, prior CSC treatments, and hyperreflective material on OCT.

**Results:**

Thirty-seven patients (40 eyes; mean age, 48.0 ± 11.9 years) had CSC and BALAD and were followed for a mean of 4.92 ± 6.65 months. The control group was comprised of 40 patients with CSC without BALAD (40 eyes; mean age, 48.2 ± 11.9 years). On clinical examination, BALAD was as a circular, yellowish macular lesion. On OCT, BALAD was a detachment of the ellipsoid zone with splitting of the photoreceptor inner segment. BALAD was associated with psychiatric disorders (*p* = 0.014), use of corticosteroids (*p* = 0.004), previous treatment for CSC (*p* = 0.041) and thickened choroid (*p* = 0.036).

**Conclusions:**

BALAD in CSC differs from a typical CSC due to the presence of a circular, yellowish macular lesion, detachment of the ellipsoid zone, segmentation of the inner segment of the photoreceptor, a thicker choroid, the use of corticosteroids, and generally more aggressive previous treatments. These results suggest that BALAD may serve as a valuable biomarker for the severity of CSC and highlight the influence of inflammation and previous treatments.

## Introduction

Central serous chorioretinopathy (CSC) is a multifactorial disease characterized by neurosensory macular detachment, subretinal fluid accumulation, retinal pigment epithelium (RPE) detachment, increased choroidal hyperpermeability and thickness, and sudden central visual loss in the acute form [1–6].

Bacillary layer detachment (BALAD) is a distinct retinal condition characterized by the separation of the inner segments of the photoreceptors resulting from the accumulation of intraretinal fluid between the myoid and ellipsoid zones in optical coherence tomography (OCT) [1–24]. It was firstly noted in 2018, when Mehta et al. [16] describe this separation in case of macular toxoplasmosis chorioretinitis in OCT images. This detachment occurs by damage to the outer retina and is confined to the space between the external limiting membrane (ELM) and the junctional complexes of the retinal pigment epithelium (RPE) [3, 5, 8–25]. BALAD has been observed in various conditions, including Vogt-Koyanagi-Harada disease, sympathetic ophthalmia, uveal effusion syndrome, acute posterior multifocal placoid pigment epitheliopathy, age-related macular degeneration, ocular trauma, and CSC [1–10].

To date, the incidence of BALAD in CSC is still unknown. This study aims to describe the clinical characteristics and identify risk factors associated with central serous chorioretinopathy and bacillary detachment, as observed in various centers worldwide.

## Methods

A retrospective case–control study was conducted across 12 retina centers worldwide over a 4-month period from December 1, 2022, to April 1, 2023, with the aim of identifying patients diagnosed with CSC and Bacillary layer detachment (BALAD). The study was conducted in accordance with the Declaration of Helsinki, and written informed consent was obtained from all participants. Institutional Review Board approval was provided at each participating center.

CSC diagnosis was based on the presence of serous detachment of the neurosensory retina through ophthalmoscopy and optical coherence tomography (OCT). Common symptoms among patients included distorted central vision, metamorphopsia, reduced visual acuity, and the presence of a dark spot in central visual field.

Patients were included in the study if they met the following inclusion criteria: (1) CSC diagnostic based on the presence of a neurosensory detachment, one or more sites of leakage from the RPE, and absence of other causes of exudation, such as choroidal neovascularization or primary choroidal inflammation; (2) presence of BALAD [3], identified by the collaborators and confirmed by three authors (AMBC, RF and LHL); (3) age over 18 years; and (4) a detailed medical history including concomitant diseases and medication use within 30 days prior to the onset of CSC symptoms.

Patients were excluded if they had: (1) retinal vascular disorders (e.g., hypertensive retinopathy, diabetic retinopathy, retinal artery occlusion, or retinal vein occlusion); (2) inflammatory disorders affecting the posterior pole; (3) congenital cardiovascular diseases; and (4) significant media opacities (e.g., cataract or corneal opacity).

BALAD was identified based on its morphology, characterized by the separation of the bacillary layer in the foveal or parafoveal regions. On OCT imaging, the detachment was characterized by a hyporeflective space located immediately posterior to the external limiting membrane (ELM). The upper limit of this space was marked by a granular line with variable hyper-reflectivity, while the base or posterior limit was indicated by a line of variable reflectivity and thickness. This line extended from the ellipsoid zone of the adjacent retina, connecting to the basal lamina of the RPE and Bruch's membrane [3, 11, 17–24].

All patients underwent comprehensive ophthalmologic evaluation, which included: best-corrected visual acuity (BCVA) assessment, slit-lamp biomicroscopy, tonometry, indirect fundus ophthalmoscopy, color fundus photography, spectral-domain optical coherence tomography (OCT) (Cirrus 6000, Zeiss, Dublin, California or Spectralis, Heidelberg Engineering, Germany), and swept-source OCT (Triton, Topcon Corporation, Tokyo, Japan). Choroidal thickness was measured in the subfoveal region, and the height of BALAD was measured from the ELM to the RPE. Additionally, fluorescein angiography (FA) (HRA, Heidelberg Engineering, Heidelberg, Germany), indocyanine green angiography (ICGA) (HRA, Heidelberg Engineering, Heidelberg, Germany) and fundus autofluorescence (FAF) (Clarus 700, Zeiss, Dublin, California, Triton, Topcon Corporation, Tokyo, Japan or HRA, Heidelberg Engineering, Heidelberg, Germany), were performed on 15 eyes from the study cases.

The study case group consisted of individuals with both CSC and BALAD, while the control group included individuals with CSC but without BALAD. Control group cases were collected from the same 12 retina centers and were matched with the study control by age, gender, and type of CSC. CSC cases were categorized into acute and chronic forms. Acute CSC was defined as a single, sudden episode of fluid leakage from the choroid into the subretinal space, while chronic CSC involved recurrent or persistent episodes of subretinal fluid leakage over time.

Variables analyzed included BCVA, history of anxiety or stress disorder, systemic hypertension or coronary artery disease, corticosteroid use (in all forms), previous therapy for CSC, COVID-19 infection, and subfoveal choroidal thickness (SFCT). BCVA values were converted to the logarithm of the minimum angle of resolution (logMAR) for analysis. SFCT measurements were performed by collaborators and rechecked by three authors (AMBC, RF and LHL).

Statistical analyses were conducted using R software, version 4.1.1 [30, 31]. Non-probability and non-random sampling methods were employed in the study. Quantitative variables were analyzed using means, percentages, and standard deviations. For normally distributed variables, the Student’s t-test was used to compare means between the case and control groups. For non-normally distributed variables, the Mann–Whitney U test was applied. Descriptive analyses were presented as numbers and percentages for categorical variables, and as means ± standard deviations for quantitative variables. The chi-square test or Fisher’s exact test was used for qualitative variables when appropriate. Comparisons of BCVA between diagnosis and follow-up were made using the Student’s t-test or Wilcoxon signed-rank test, as appropriate. A p-value < 0.05 was considered statistically significant.

To estimate the prevalence of BALAD within the CSC population, three collaborators from different continents (North America, South America, and Asia) were asked to review their records over the study period (December 1, 2022, and April 1, 2023). All images were meticulously reviewed by three authors (AMBC, RF and LHL).

## Results

**Fig. 1:**
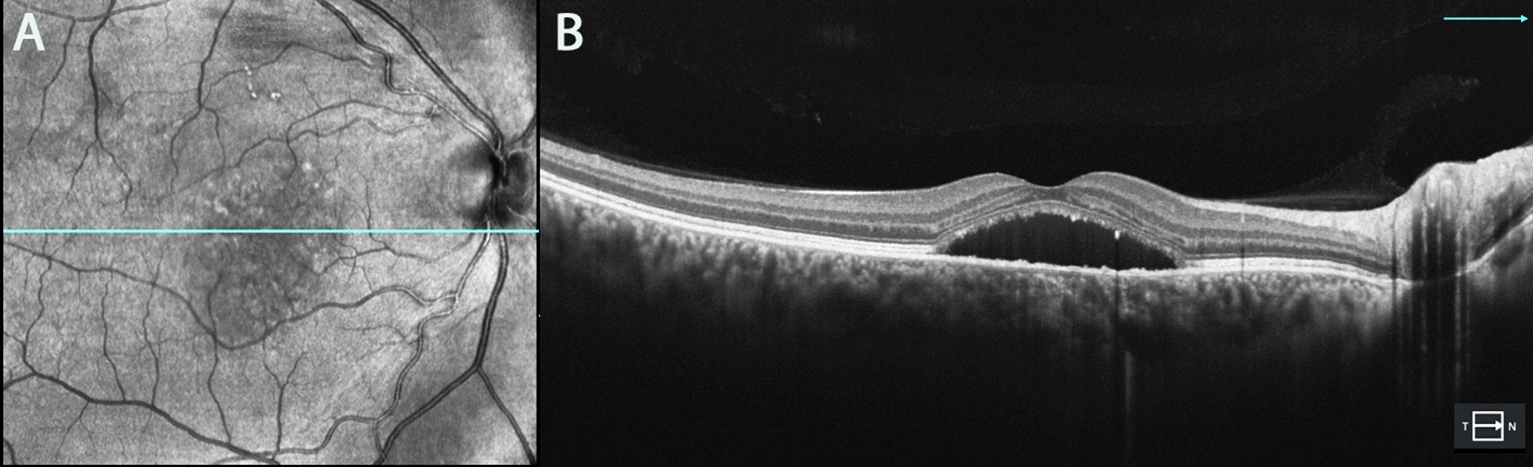
Optical coherence tomography images (**A**, **B**) of a 54-year-old patient in the control group with chronic CSC

The study group included 37 individuals (40 eyes) with CSC and BALAD, and the control group included 40 individuals (40 eyes) with CSC without BALAD. In the BALAD group, there was a predominance of male patients (75.7%) between 40 and 50 years of age (34.2%), unilateral retinal involvement (92.1%) and chronic CSC (82.5%) (Fig. 1).

**Fig. 2:**
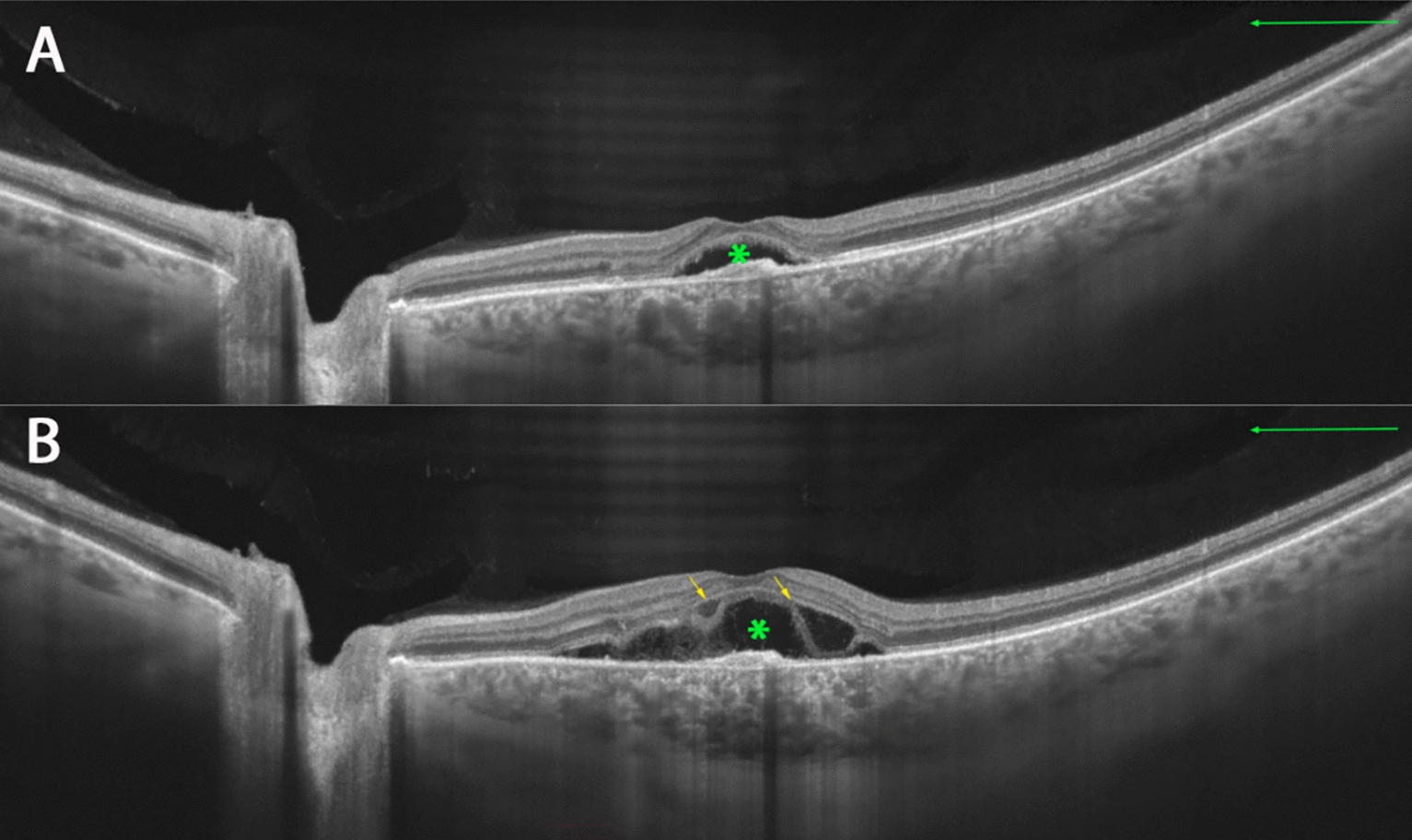
Optical coherence tomography (**A**, **B**) of a patient with chronic CSC and BALAD. Patient had low vision shortly after receiving treatment with photodynamic therapy. The yellow arrow represents BALAD, and the green asterisk indicates SRF. *BALAD* bacillary layer detachments, *SRF* subretinal fluid

**Fig. 3:**
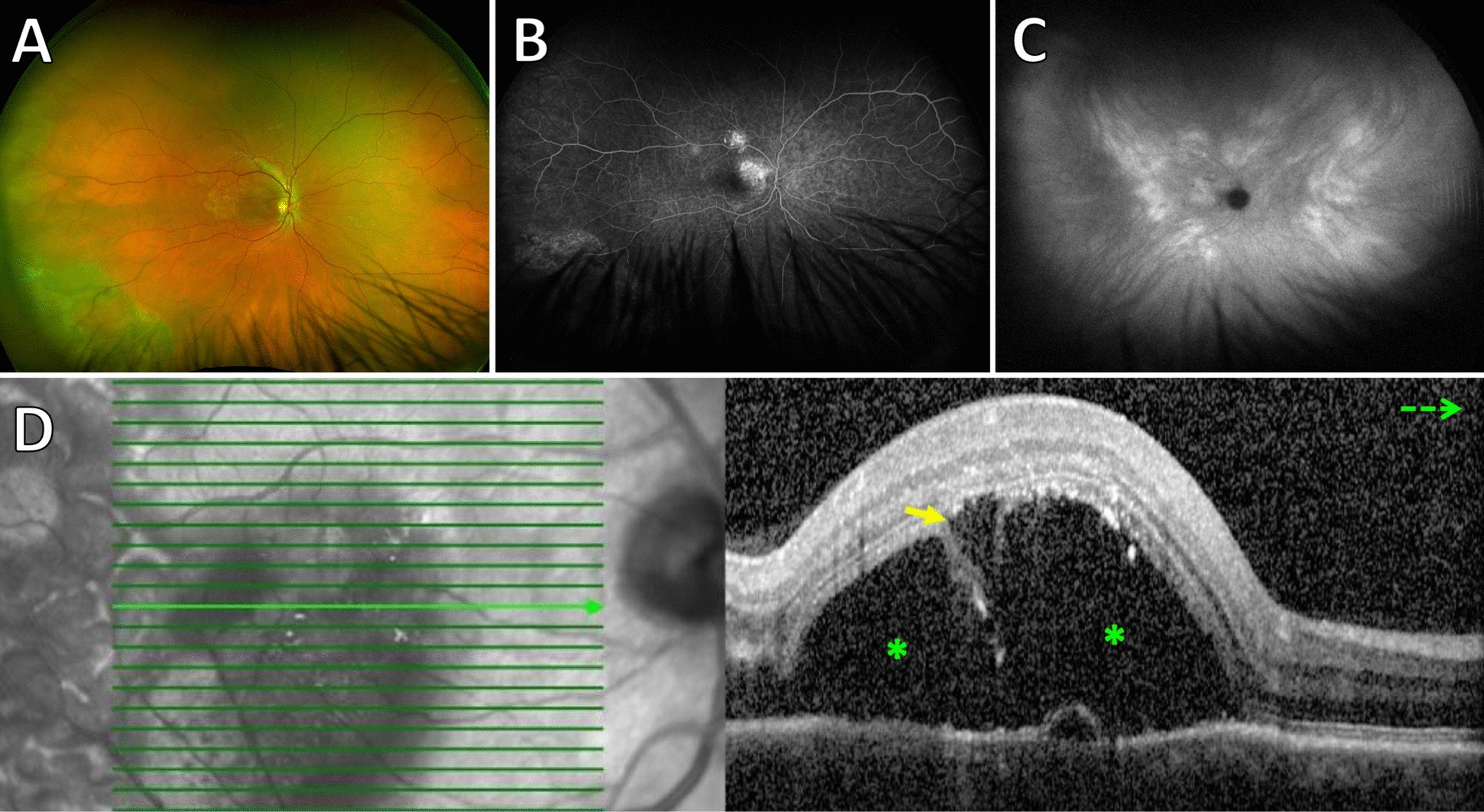
Fundus photograph (**A**), fluorescein angiography (**B**), indocyanine green angiography (**C**) and optical coherence tomography (**D**) of a 58-year-old male patient who developed BALAD after treatment of chronic CSC by photodynamic therapy. Optical coherence tomography angiography (OCTA) excluded choroidal neovascularization. The yellow arrow represents BALAD, and the green asterisk indicates SRF. *BALAD* bacillary layer detachments, *SRF* subretinal fluid

**Fig. 4:**
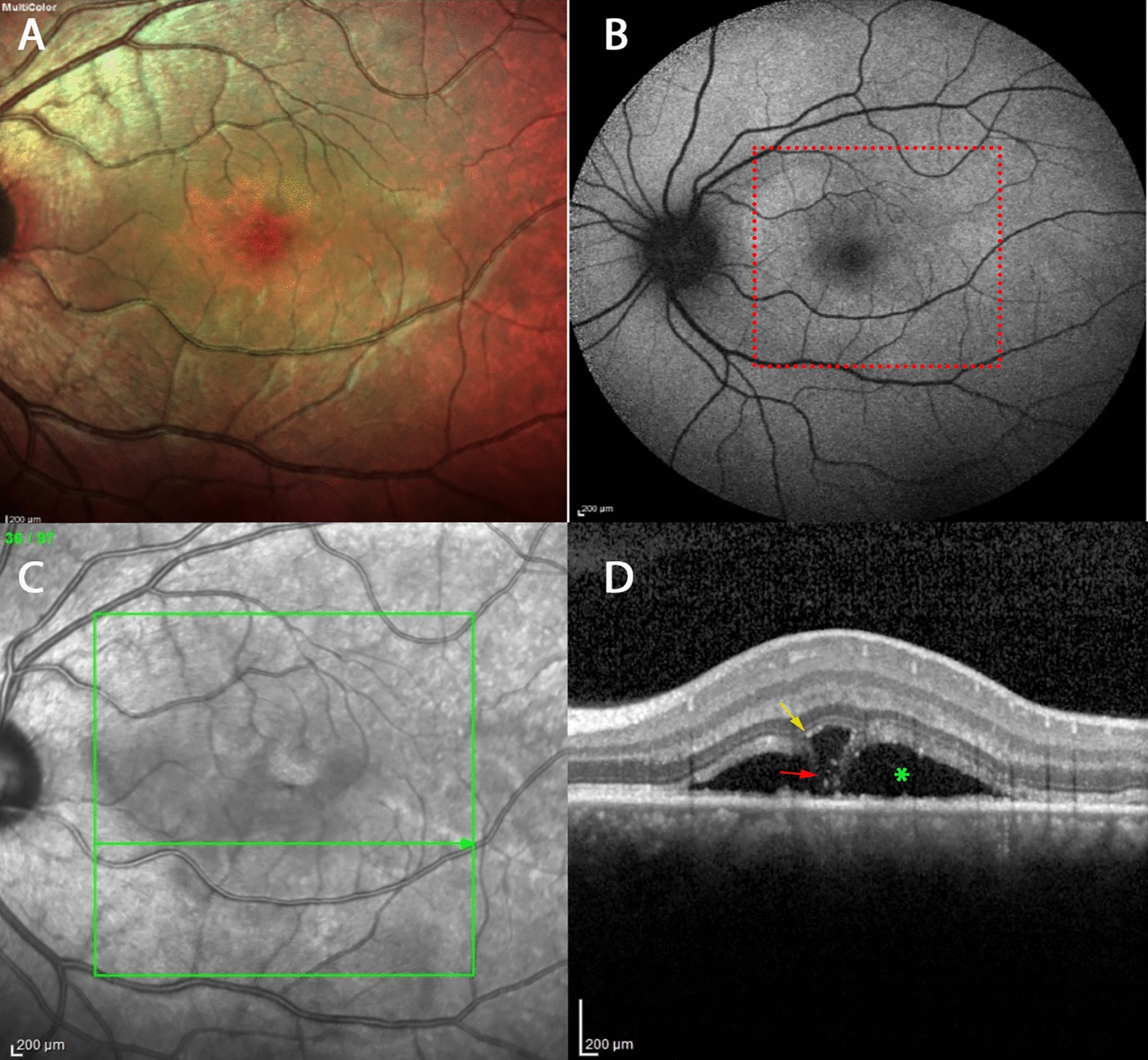
Fundus photograph (**A**), Autofluorescence (**B**), and optical coherence tomography images (**C** and **D**) of the left eye of a 26-year-old man with acute bilateral central serous chorioretinopathy with BALAD. Images show the presence of hyper-reflectivity within the BALAD cavity (red arrow). The yellow arrow represents BALAD, and the green asterisk indicates SRF. The red arrow represents materials with hyperreflectivity. *BALAD* bacillary layer detachments, *SRF* subretinal fluid

**Fig. 5:**
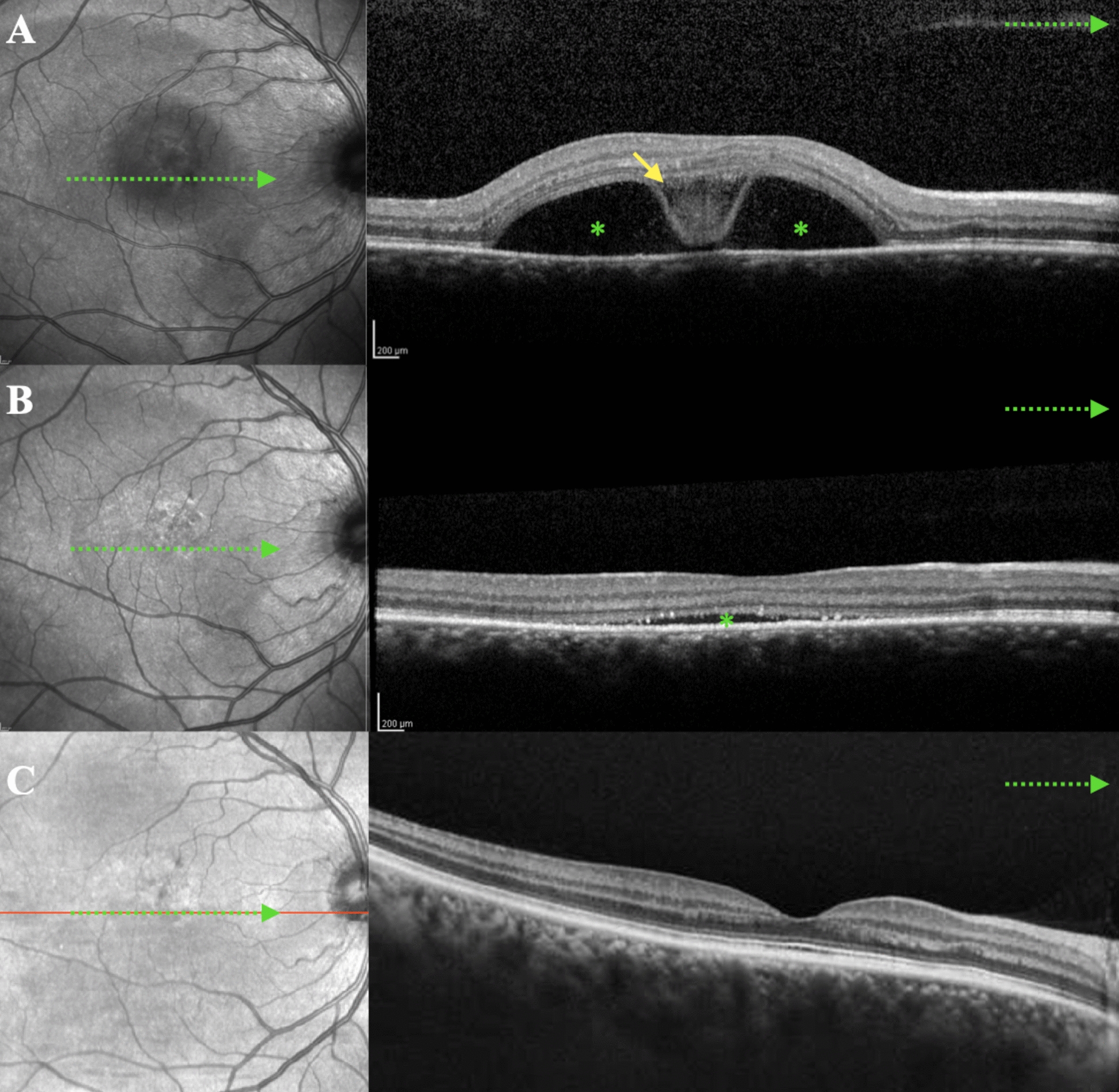
Sequence of OCT images of a 43-year-old woman with acute CSC and BALAD in the right eye. **A** 20 days after COVID-19 diagnosis and corticosteroid use. **B** 30-day follow-up after stopping corticosteroids. **C** 60-day follow-up showing resolution. The yellow arrow indicates BALAD, and the green asterisk represents subretinal fluid (SRF). The yellow arrow represents BALAD, and the green asterisk indicates SRF. *BALAD* bacillary layer detachments, *SRF* Subretinal fluid

**Fig. 6:**
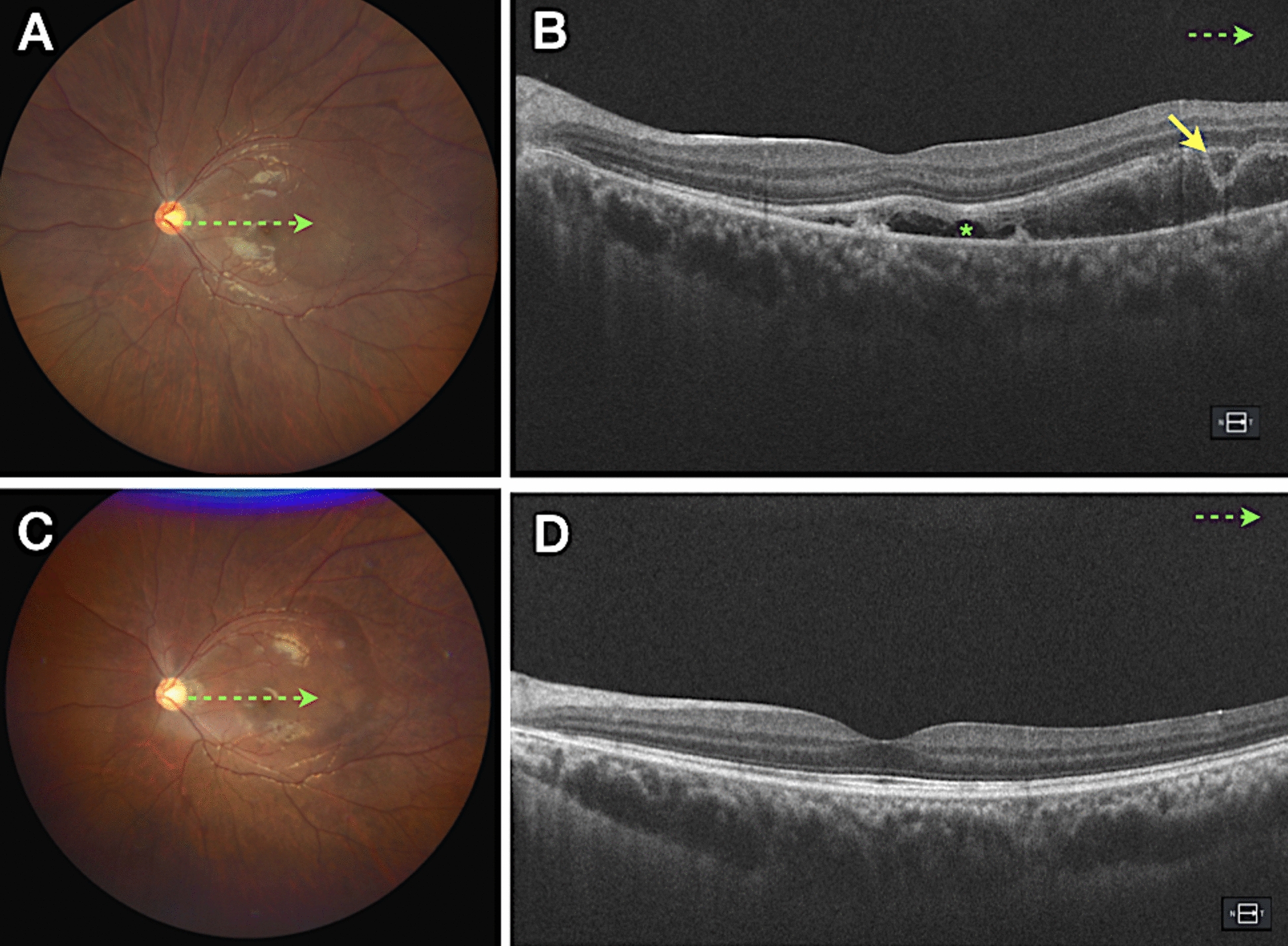
Fundus photographs (**A**, **C**) and optical coherence tomography (OCT) images (**B**, **D**) of a 22-year-old male who presented with low vision 28 days after developing acute central serous chorioretinopathy (CSC), following treatment with high doses of oral corticosteroids for systemic lupus erythematosus. It is likely that corticosteroid use exacerbated the serous retinal detachments, leading to vascular compromise that affects the integrity of the retinal pigment epithelium (RPE) and neurosensory retina, promoting the onset of CSC. Images (**C**) and (**D**) show the patient at a 30-day follow-up. The yellow arrow indicates Bacillary Layer Detachment (BALAD), and the green asterisk marks subretinal fluid (SRF). *BALAD* Bacillary Layer Detachments, *SRF* Subretinal Fluid

The comparison between CSC and BALAD patients to CSC controls showed a strong correlation with psychiatric disease (p = 0.014), use of corticosteroids (p = 0.004), previous treatment for CSC (p = 0.041) (Figs. 2 and 3), and more significant increase in choroidal thickness (p = 0.036) (Table 1). The eyes with CSC with BALAD had distinct round yellowish foveal lesion within the neurosensory retinal detachment (Figs. 1, 2, 3, 4, 5, 6) and greater choroidal thickness than control eyes (448 ± 105.6 µm vs. 388.0 ± 82.0 µm).

**Table 1:** Characteristics of the Two Study Groups Classified According to the Presence of a BALAD (Case Group) and no BALAD (Control Group) in Patients with CSC eyes

Variable	Cases (n, % or Mean ± SD)	Controls (n, % or Mean ± SD)	*P* Value
Age (years)	48.0 ± 11.9	48.2 ± 11.9	0.807*
Gender			0.945†
Male	28 (75.7)	30 (75)	
Female	9 (24.3)	10 (25)	
Duration of symptoms			1.000†
aCSC	7 (17.5)	7 (17.5)	
cCSC	33 (82.5)	33 (82.5)	
Systemic disease			
Hypertension or CAD	12 (30)	8 (20)	0.302†
Psychiatric disorders	13 (32.5)	4 (10)	**0.014**†
Use of corticosteroids	15 (37.5)	4 (10)	**0.004**†
COVID-19 positivity	4 (9.8)	None	0.116‡
Previous treatment for CSC	21 (52.5)	12 (30)	**0.041**†
Photodynamic therapy	12 (57.1)	5 (50.0)	
PDT and anti-VEGF	1 (4.8%)	None	
IVT anti-VEGF therapy	3 (14.3)	1 (10)	
Mineralocorticoid antagonist	2 (9.5)	1 (10)	
Mineralocorticoid antagonist and LA	None	None	
LA	2 (9.5)	3 (30)	
LA and anti-VEGF	1 (4.8)	None	
Treatment for CSC (follow-up)	14 (35)	11 (27.5)	
Photodynamic therapy	3 (21.4)	None	
PDT and anti-VEGF	1 (7.1)	None	
IVT anti-VEGF therapy	2 (14.3)	3 (27.3)	
Mineralocorticoid antagonist	1 (7.1)	2 (18.2)	
Mineralocorticoid antagonist and LA	1 (7.1)	None	
LA	4 (28.6)	6 (54.5)	
LA and anti-VEGF	2 (14.3)	None	0.469‡
Clinical characteristics			
Initial BCVA (logMAR)	0.473 ± 0.39	0.390 ± 0.32	0.908**
Final BCVA (logMAR)	0.447 ± 0.30	0.341 ± 0.17	0.186**
OCT			
Choroidal thickness (mean ± SD)	448.7 ± 105.6	388.0 ± 82.0	0.036*
Fibrin present	13 (34.2)	11 (44)	0.434†

*BALAD* bacillary layer detachment, *CSC* central serous chorioretinopathy, *aCSC* acute central serous chorioretinopathy, *cCSC* chronic central serous chorioretinopathy, *SD* standard deviation, *CAD* cardiovascular diseases, *VEGF* vascular endothelial growth factor, *IVT* intravitreal therapy, *OCT* optical coherence tomography, *PDT* photodynamic therapy, *COVID-19* coronavirus disease, *BCVA* best-corrected visual acuity baseline, laser photocoagulation (*LA*)

^*^Student’s test

^†^Chi-square test

^‡^Fisher exact test

^**^Mann–Whitney U test

In the BALAD group, the mean duration of visual symptoms was 186 ± 202 days and the mean follow-up was 4.92 ± 6.65 months (median, 3 months; range, 35 (1–36 months). During follow-up, OCT images confirmed the progressive and gradual complete resolution of the subretinal fluid and the BALAD to complete resolution (Figs. 5 and 6). The mean height of BALAD was 370 ± 274 µm (median: 279 µm; range: 110–1054 µm).

The improvement in visual acuity between the initial and final evaluations was noted in both groups (Student’s t: 2.76, df: 23, p = 0.011 vs. Wilcoxon W = 347, p = 0.005 vs Wilcoxon W = 306, p = 0.005); however, the BALAD patients generally had more severe CSC initially. The mean baseline BCVA was 20/59 (0.473 ± 0.39 logMAR; median, 0.495 logMAR; range, 0–1.69 logMAR) and throughout the follow-up period, change in final BCVA (0.447 ± 0.30 logMAR; median, 0.300 logMAR; range, 0.00–1.69 logMAR (Table 1).

Four patients were diagnosed positive for COVID-19 via PCR and developed BALAD. These patients exhibited characteristics, and a history of corticosteroid use during or after the infection. Case 1 involves a 41-year-old male who experienced vision loss in his left eye 13 days after contracting SARS-CoV-2 and starting oral dexamethasone. Case 2 describes a 43-year-old female who developed vision loss in her right eye 20 days after testing positive for COVID-19, following treatment for pneumonia with high doses of intravenous and oral dexamethasone. In Case 3, a 49-year-old male with a history of CSC developed reduced vision in his right eye 30 days after COVID-19 infection and the use of prednisone. Case 4 involves a 44-year-old Caucasian male who experienced vision loss 40 days after contracting COVID-19 and using high-dose oral corticosteroids.

In the BALAD group, 4 (10%) had hypermetropia compared to 12 (30%) in the control group, 11 (27.5%) were emmetropes compared to 11 (27.5%), and 4 (10%) had myopia compared to 10 (25%). In 21 (52.5%) cases, refraction testing was either not performed or not recorded, compared to 17.5% in the control group (p = 0.003).

The prevalence of BALAD in CSC populations in 3 tertiary referral centers varied from 2.5% in North America, 5.0% in South America, and 5.8% in Asia (respectively 1:40; 7:140 and 7:120).

## Discussion

This case–control study evaluated 37 patients with CSC and BALAD and 40 patients with CSC without BALAD. The findings highlight several significant differences between these two groups, especially with the clinical characteristics and associated conditions.

One of the most significant findings was the strong correlation between BALAD and various systemic conditions, particularly psychiatric disorders (32.5% vs. 10%, p = 0.014) and corticosteroid use (37.5% vs. 10%, p = 0.032), both established risk factors for CSC. These associations suggest that BALAD may represent a more severe or complex form of CSC, potentially influenced by systemic inflammation or drug effects. Although the pathogenesis is still unclear, BALAD may result from intense force on the myoid zone of the photoreceptor inner segment to exceed the limits, leading to detachment during intense and acute subretinal exudation [3, 6, 8, 13–24].

In predisposed individuals, such as those with a psychiatric disorders and type A personality, catecholamine release can increase choroidal vessel permeability, disrupting their functional balance. Evidence also suggests that stress contributes to both the development and worsening of CSC. Stress-induced sympathetic stimulation tends to increase choroidal blood flow, promoting the onset and progression of the condition. This increase in flow may lead to focal choroidal ischemia, damaging the RPE and impairing its function. Localized ischemia can, in turn, induce greater vascular exudation, contributing to the development of serous pigment epithelial detachment (PED) and compromising the outer blood-retinal barrier, thereby intensifying the clinical presentation of CSC [28, 29].

Additionally, BALAD predominantly developed unilaterally (91.4%), often localized in the foveal or parafoveal regions, and typically occurred as a single focal lesion. OCT imaging revealed distinctive features in BALAD group, including a round, yellowish foveal lesion within the neurosensory retinal detachment and increased subfoveal choroidal thickness (SFCT).

The BALAD group was notably associated with visual decline due to subretinal fluid, although there was a significant improvement in BCVA between the initial and final evaluations (Student’s t-test: 2.76, df: 23, p = 0.011; Wilcoxon test: W = 347, p = 0.005 for the control group). Initially, the BALAD group presented with a more severe form of CSC, which may have contributed to worse functional outcomes at baseline. However, both groups demonstrated significant improvements in visual acuity during the follow-up period. Further information regarding treatment requirements and long-term changes in the retinal pigment epithelium (RPE) would be crucial for a more comprehensive understanding of treatment burden and the potential for atrophy.

Consistent with our findings, visual deterioration in eyes with BALAD has previously been reported in patients with age-related macular degeneration [18, 23, 24] and has been identified as a transient condition [18, 25], as corroborated by our results. These anatomical improvements were observed with the resolution of CSC, likely due to increased choroidal blood flow and improved metabolic support for the photoreceptor inner segments provided by mitochondria [3, 10, 11, 15]. Furthermore, the rapid regenerative capacity of the inner and outer segments of the photoreceptors [3] is likely a contributing factor to this improvement.

Notably, hyper-reflective particles were observed suspended within the BALAD cavity in thirteen cases (34.2%). These suspended hyper-reflective particles are often related with BALAD [24]. While alternative explanations could not be definitively excluded, we propose that these hyper-reflective materials may represent fibrin, photoreceptor debris, septa or even mitochondrial aggregates [3, 8, 12, 14, 17–19, 22].

SFCT was significantly greater in the BALAD group (448 ± 105.6 µm vs. 388.0 ± 82.0 µm in the control group, p = 0.036), reinforcing the hypothesis that choroidal changes play a central role in the pathophysiology of BALAD in CSC. This thickening may be attributed to choroidal ischemia [3, 11–15] and choroidal inflammation [24], which are known to compromise the outer blood-retinal barrier. These changes have been documented in association with inflammatory or infiltrative retinopathies [17, 25], trauma [17], acute posterior multifocal placoid pigment epitheliopathy [12, 17], and pachychoroid-associated serous chorioretinopathy [17], and could potentially lead to BALAD.

Choroidal ischemia reduces the perfusion of the outer retina, increases hydrostatic pressure, and compromises the integrity of the outer blood-retinal barrier by damaging the RPE. This leads to nutritional stress on the bacillary layer, which relies on the choriocapillaris for nourishment and regeneration [3, 11–15]. In BALAD, these effects likely occur more abruptly, rapidly, and intensely. As observed in our study, this has been resolved concurrently with the resolution of inflammation and fluid accumulation [24].

We hypothesize that an intense inflammatory process and prior treatments, whether through direct or indirect injury, play a key role in the pathophysiology of BALAD. The development of BALAD may be linked to severe, acute choroidal exudation into the subretinal space, which stretches and surpasses the tensile resistance of the photoreceptor inner segments, leading to splitting of the myoid layer and its detachment from the ellipsoid zone. Chronic CSC may further predispose photoreceptors to BALAD, largely due to apoptosis of the photoreceptor outer segments. This process results from the chronic accumulation of subretinal fluid, which triggers oxidative stress and inflammation, weakening the adhesion between the RPE and photoreceptors. Such weakening can result in layer detachment as photoreceptor degeneration progresses within an inflammatory environment. Similar effects have been observed in cases following photodynamic therapy (PDT), high hyperopia, and COVID-19 infection, [7–10] where the occurrence of BALAD has been linked to severe local inflammatory reactions induced by PDT, systemic inflammation associated with COVID-19, and choroidal thickening [1–10].

Although we did not specify the treatments administered during the study period, we observed a higher proportion of patients who had previously received treatments for CSC, including photodynamic therapy (PDT) (p = 0.041; 12 out of 21 eyes were treated with PDT). PDT is commonly used in CSC management as it selectively occludes abnormal choroidal vessels, reducing fluid leakage. However, PDT is also known to provoke intense local inflammatory reactions, which may contribute to BALAD development. Fernández-Vigo et al. [7] reported BALAD in 13 out of 98 CSC patients treated with PDT, supporting its potential link to inflammatory processes. Although there is no direct evidence that PDT damages mineralocorticoid receptors (MRs), recently identified in the choroid, retinal pigment epithelium (RPE), and photoreceptors, it may indirectly affect these receptors. PDT-induced inflammation and oxidative stress could alter MR expression or sensitivity. MRs play a crucial role in choroidal water balance and vascular pressure regulation and contribute to choroidal thickening. This suggests that MRs may be vital in modulating choroidal blood flow and vascular permeability, with potential implications for retinal homeostasis and CSC pathology [26–28].

Similarly, patients with high hyperopia often have a thicker sclera, which can cause fluid accumulation in the choroid due to increased resistance along the vortex vein pathway, like uveal effusion syndrome. The vortex vein crosses a longer intra-scleral path, increasing the risk of blockage and outflow resistance. This thicker sclera reduces permeability and intensifies venous resistance, raising local pressure and fluid buildup. In our study, a high rate of missing exams in the case group (52.5%) compared to controls (17.5%) may bias results, potentially affecting interpretation of the association between ametropias and the condition studied [26, 27, 29].

In patients with COVID-19, exacerbated interleukin activation leads to a cytokine storm due to intense systemic inflammatory manifestations. Additionally, these patients received high doses of systemic corticosteroids, which, combined with the inflammatory state caused by the infection, may contribute to the unique characteristics observed in the disease. These cases suggest a potential association between COVID-19 infection, corticosteroid use, and the development of BALAD in susceptible individuals. People with pro-thrombotic conditions, such as COVID-19 and systemic lupus erythematosus (SLE), may experience compression of the choriocapillaris and Sattler’s layer, resulting in RPE dysfunction. This leads to the accumulation of large molecules, like fibrin, in the subretinal space, which may cause detachment and other RPE alterations [3, 6, 10, 13, 17, 22].

This study has several limitations, including its retrospective design, relatively small sample size, and lack of long-term follow-up data. The limited sample of 37 patients with BALAD impacts the statistical robustness and generalizability of the findings. With a smaller sample, there is a higher risk of bias and reduced statistical power, which makes it challenging to detect subtle associations or differences between variables. This may result in findings that are not fully representative of the broader population, thereby limiting the reliability of the conclusions. Furthermore, individual variability has a greater influence in smaller samples, meaning that individual differences can disproportionately impact the results compared to larger samples, where such variations tend to balance out. To mitigate these limitations, we implemented adjustments to control for potential confounding factors by matching cases with controls of similar age and disease severity. These adjustments improve the precision of our estimates and the internal validity of the study, making the findings more reliable despite the sample size limitation.

Another limitation is the lack of data on axial length and refractive status for all eyes, which may hinder our assessment of the relationship between CSC and BALAD (bacillary layer detachment). Factors such as hyperopia can alter choroidal circulation dynamics, potentially influencing susceptibility to BALAD. Without this information, it is more challenging to fully understand the role that these anatomical variables play in BALAD development in CSC patients, limiting our ability to determine if BALAD occurs more frequently in specific ocular profiles.

Although, to our knowledge, this case–control study appears to be the largest in the literature concerning the rare disease of CSC with BALAD (PubMed search, July 2024).

In conclusion, the BALAD group highlights the significant role of inflammation in the association with CSC, suggesting that BALAD could serve as a potential biomarker, even though the precise role in chronic CSC remaining unclear. The findings suggest that the BALAD group experienced a more severe condition, characterized by a higher incidence of comorbidities, more aggressive or ineffective prior treatments, and less favorable initial clinical characteristics. These results reveal distinct ocular changes, broadening the spectrum of CSC presentations and enabling differentiation from other similar macular diseases. Additionally, this study emphasizes several clinical implications, including notable choroidal thickening and significant correlations with corticosteroid use, psychiatric disorders, and prior treatments, particularly photodynamic therapy (PDT).

## Data Availability

No datasets were generated or analysed during the current study.

## Abbreviations

CSC: Central serous chorioretinopathy
BALAD: Bacillary layer detachment
PDT: Photodynamic therapy
RPE: Retinal pigment epithelium
FA: Fluorescein angiography
BCVA: Best-corrected visual acuity
OCT: Optical coherence tomography
SFCT: Subfoveal choroidal thickness
